# Robust evidence for bats as reservoir hosts is lacking in most African virus studies: a review and call to optimize sampling and conserve bats

**DOI:** 10.1098/rsbl.2023.0358

**Published:** 2023-11-15

**Authors:** Natalie Weber, Martina Nagy, Wanda Markotter, Juliane Schaer, Sébastien J. Puechmaille, Jack Sutton, Liliana M. Dávalos, Marie-Claire Dusabe, Imran Ejotre, M. Brock Fenton, Mirjam Knörnschild, Adrià López-Baucells, Rodrigo A. Medellin, Markus Metz, Samira Mubareka, Olivier Nsengimana, M. Teague O'Mara, Paul A. Racey, Merlin Tuttle, Innocent Twizeyimana, Amanda Vicente-Santos, Marco Tschapka, Christian C. Voigt, Martin Wikelski, Dina K.N. Dechmann, DeeAnn M. Reeder

**Affiliations:** ^1^ Department of Migration, Max Planck Institute of Animal Behavior, Radolfzell, Germany; ^2^ University of Ulm, Institute of Evolutionary Ecology and Conservation Genomics, Ulm, Germany; ^3^ Museum für Naturkunde, Leibniz-Institute for Evolution and Biodiversity Science, Berlin, Germany; ^4^ Centre for Viral Zoonoses, Department of Medical Virology, Faculty of Health Sciences, University of Pretoria, Pretoria, South Africa; ^5^ Institute of Biology, Humboldt University, Berlin, Germany; ^6^ ISEM, University of Montpellier, Montpellier, France; ^7^ Institut Universitaire de France, Paris, France; ^8^ Zoological Institute and Museum, University of Greifswald, Greifswald, Germany; ^9^ Bucknell University, Lewisburg, PA, USA; ^10^ Department of Ecology and Evolution and Consortium for Inter-Disciplinary Environmental Research, Stony Brook University, Stony Brook, USA; ^11^ Rwanda Wildlife Conservation Association, Kigali, Rwanda; ^12^ Muni University, Arua, Uganda; ^13^ Department of Biology, University of Western Ontario, London, Ontario, Canada; ^14^ Evolutionary Ethology, Institute for Biology, Humboldt-Universität zu Berlin, Berlin, Germany; ^15^ Smithsonian Tropical Research Institute, Balboa, Ancón, Panama; ^16^ BiBio Research Group, Natural Science Museum of Granollers, Granollers, Spain; ^17^ Institute of Ecology, National Autonomous University of Mexico, Mexico City, Mexico; ^18^ mundialis GmbH & Co. KG, Bonn, Germany; ^19^ Sunnybrook Research Institute and Department of Laboratory Medicine and Pathobiology, University of Toronto, Toronto, Ontario, Canada; ^20^ Bat Conservation International Austin, TX, USA; ^21^ Department of Biological Sciences, Southeastern Louisiana University, Hammond, LA, USA; ^22^ Centre for Ecology and Conservation, University of Exeter, Exeter, UK; ^23^ Merlin Tuttle's Bat Conservation, Austin, TX USA; ^24^ Department of Integrative Biology, University of Texas, Austin, USA; ^25^ Graduate Program in Population Biology, Ecology and Emory University, Atlanta, GA, USA; ^26^ Department of Biology, University of Oklahoma, Norman, OK, USA; ^27^ Leibniz Institute for Zoo and Wildlife Research, Berlin, Germany; ^28^ Department of Biology, University of Konstanz, Konstanz, Germany

**Keywords:** African Chiroptera, virus–host relationship, virological metadata, framing, One Health

## Abstract

Africa experiences frequent emerging disease outbreaks among humans, with bats often proposed as zoonotic pathogen hosts. We comprehensively reviewed virus–bat findings from papers published between 1978 and 2020 to evaluate the evidence that African bats are reservoir and/or bridging hosts for viruses that cause human disease. We present data from 162 papers (of 1322) with original findings on (1) numbers and species of bats sampled across bat families and the continent, (2) how bats were selected for study inclusion, (3) if bats were terminally sampled, (4) what types of ecological data, if any, were recorded and (5) which viruses were detected and with what methodology. We propose a scheme for evaluating presumed virus–host relationships by evidence type and quality, using the contrasting available evidence for Orthoebolavirus versus Orthomarburgvirus as an example. We review the wording in abstracts and discussions of all 162 papers, identifying key framing terms, how these refer to findings, and how they might contribute to people's beliefs about bats. We discuss the impact of scientific research communication on public perception and emphasize the need for strategies that minimize human–bat conflict and support bat conservation. Finally, we make recommendations for best practices that will improve virological study metadata.

## 1. Introduction

Viral spillover from wildlife to humans is a global threat [1–3]. Despite their significance, identification of reservoir hosts, transmission mechanisms and conditions, and pathogenicity remain unknown for most viruses. Several human diseases hypothesized to have originated in bats have devastating effects, as exemplified by the 2019–2023 ongoing COVID-19 pandemic and re-emerging Ebola virus outbreaks [4–7]. Intense, ongoing global surveillance for bat viruses is generating a rapidly growing body of the literature [8]. However, heterogeneity of field and laboratory methods, a paucity of data on bat biology and ecology, and a lack of surveillance in other mammalian groups that may play a role in spillover, have limited reliable assignment of reservoir host status (bat and otherwise) and hamper our understanding of complex multi-host transmission and spillover dynamics.

Many outbreaks of emerging diseases occur in Africa, which has a unique, diverse and ecologically important assemblage of bat species [9,10]. Unlike the well-characterized and well-known Australian and SE Asian *Henipavirus*–*Pteropus* flying fox systems [11,12], and with the exception of comprehensive work on the African *Orthomarburgvirus*–*Rousettus aegyptiacus* system [13–28], the disease ecology of African bats is understudied, especially in light of African bat biodiversity and the size of the continent. Herein, we review published field studies on African bat viruses, summarizing and analysing the work published through 2020 to evaluate the types and quality of data available, trends in species and localities sampled, knowledge gaps and conservation concerns, and to make recommendations for best practices that will improve virological study metadata.

## 2. African bats and virus research

### (a) Literature review and data analysis

**Figure 1.**
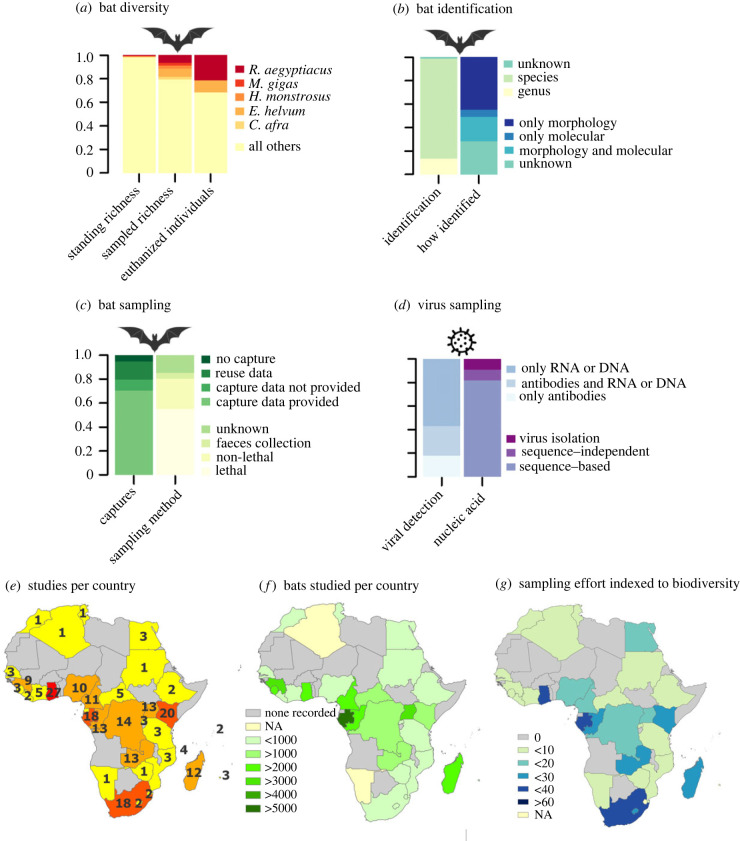
Summaries of review findings (162 papers; 1978–2020). (*a*) Bat diversity, proportion of five most frequently sampled species relative to standing species richness (first bar) and total sampling effort (out of 742 species–study combinations) (second bar), and the proportion of the two most frequently sampled species being euthanized (third bar). (*b*) Bat identification, proportion of samples where bats were identified to the genus or species level, or not at all (first bar), and whether morphology and/or molecular methods were used (second bar). (*c*) Bat sampling, proportion of studies that did or did not provide capture data (first bar), and how captured bats were sampled: lethal, non-lethal (i.e. blood, skin, urine, oral or rectal swab), faeces (roost collected) or unknown (sample type not given) (second bar). (*d*) Virus sampling, proportion of studies by detection type (first bar), and by type of evidence (second bar). (*e*) Number of bat virological studies recorded from each African country. (*f*) Number of bats recorded to have been studied by country (number of bats in Algeria and Namibia not available, numbers are nearest estimates as some studies included more than one country and did not delineate provenance). (*g*) Viral sampling efforts normalized by species-level bat biodiversity (no. studies/no. species × 100).

We analysed data from peer-reviewed primary research articles published through 2020 for which bats were captured in Africa for viral surveillance. We used the search terms ‘bat OR bats OR Chiroptera’ AND ‘virus OR viral OR virological’ AND ‘Africa OR ‘each African country name in English or country name variant’ (electronic supplementary material, figure S1) in a Web of Science (all database) search, repeated in French, yielding a total of 1322 papers (two from French search). We also included older primary data from seven studies used in 11 modelling papers from this period. In total, 162 papers met our study inclusion criteria, published between 1978 and 2020 (electronic supplementary material, text S1). This dataset, analysed alongside our current understanding of African bat systematics and ecology, provided a snapshot in time of African bat viral research from which we were able to describe the nature of these studies in detail (figure 1). Data on (1) numbers of species and individuals sampled across bat families and the continent; (2) how bats were selected for study; (3) whether they were terminally sampled; (4) whether ecological data were recorded; and (5) which viruses were detected and with what methodology, were manually extracted. We focus on four viral families most relevant to humans: *Coronaviridae, Paramyxoviridae*, *Rhabdoviridae* and *Filoviridae*, list other viral findings, and propose a schematic approach to evaluating the quality of the evidence underlying putative bat–virus relationships, using the contrasting available evidence base for *Orthoebolavirus* versus *Orthomarburgvirus* as an example. Our findings are placed in the context of numbers of known and suspected human infections and fatalities from African zoonoses associated with bats. Finally, we review the wording in abstracts and discussions of all 162 papers. We identify several key framing terms, how these refer to findings, and how they might contribute to people's beliefs about bats. In the light of the fear of bats as sources of viral spillovers, we discuss the impact of scientific research communication on public perception and emphasize the need for strategies that minimize human–bat conflict.

### (b) Species and guilds of bats sampled and their numbers

In total, 167 bat species from 11 of 13 bat families recorded in Africa were sampled (table 1 and figure 1*a*; electronic supplementary material, figure S2), representing 742 unique species–study combinations. Thirty-six of these species (21.6%) are of conservation concern and/or data deficient by the criteria of the International Union for Conservation of Nature (IUCN) ([30]; table 1). In 118 genus–study combinations, only the genus was identified (20 genera), in 10 studies (at least some) bats remained unidentified (figure 1*b*) and 16 publications listed only virus-positive bats, suggesting that greater than 167 species were likely sampled. Based on data from 70.4% (114/162) of the studies, at least 80 241 individual bats were captured; 15 studies failed to indicate numbers of captured bats (9.3%; figure 1*c*). Almost half (48.6%; ≥ 39 018 individuals) were lethally sampled, as reported in 51.9% of papers (84/162; figure 1*c*). Twenty-two studies (12.3%) did not report the fate of all bats (19.7% or 15 817 individual bats).

**Table 1.** Overview of 167 bat species sampled in 162 virus–bat research papers from Africa through 2020. Species identifications adopted from the original studies; nomenclature follows current taxonomy. For each family, number of species sampled relative to the total number of African species in that family is listed. Additionally, taxa only identified to genus or family are indicated and sampled bats may include cryptic species. Bat species are grouped alphabetically within their taxonomic families. Data include: (1) no. of non-lethal studies for that species; (2) no. of lethal studies for that species (when vouchers known); (3) whether the species inhabits caves, either obligatory ‘+’, facultatively ‘(+) or not known ‘?+’ but most species in its taxonomic group are cave-roosting; (4) IUCN Red List category when other than Least Concern (DD, data deficient; NT, near threatened; VU, vulnerable; EN, endangered; na, not assessed). Species that were only sampled once are highlighted in bold.

bat family/bat species	no. non-lethal studies	no. lethal studies	cave	IUCN Red List category	bat family/bat species	no. non-lethal studies	no. lethal studies	cave	IUCN Red List category
**Pteropodidae (29/43 species sampled, plus: 4 genus only, 1 family only)**					**Vespertilionidae (48/115 species sampled, plus: 1 genus only, 1 family only)**				
*Casinycteris argynnis*		4			*Afronycteris helios*		3		DD
***C. ophiodon***		1		NT	*A. nana*	1	9		
*Eidolon dupreanum*	4	2	(+)	VU	*Eptesicus hottentotus*		3	(+)	
*E. helvum*	18	34		NT	*E. isabellinus*	1		(+)	
***Epomophorus anselli***	1			DD	***Glauconycteris alboguttata***		1		
*E. crypturus*	1	1			*G. argentata*		3		
***E. dobsonii***	1				*G. beatrix*	1	1		
*E. gambianus*	7	5			***G. egeria***		1		DD
*E. labiatus*^a^	4	7			***G. poensis***	1			
*E. minimus*	2				*G. variegata*	1	2		
*E. pusillus*	3	8			***Hypsugo musciculus***		1		DD
*E. wahlbergi*	1	6			*Kerivoula argentata*		2		
*Epomops buettikoferi*	6	1			***K. cuprosa***		1		DD
*E. franqueti*	5	10			*K. lanosa*	1	1		
*Hypsignathus monstrosus*	7	14			***Laephotis botswanae***		1		
***Megaloglossus azagnyi***		1			*L. capensis*		5		
*M. woermanni*^b^	1	10			***L. malagasyensis***		1		VU
*Myonycteris angolensis*^c^	4	11	(+)		***L. matroka***		1		
*M. torquata*^d^	1	11			***L. robertsi***		1		DD
*Nanonycteris veldkampii*	5	2			***L. wintoni***		1		
***Plerotes anchietae***	1			DD	*L. zuluensis*	1	3		
***Pteropus niger***		1		EN	*Mimetillus moloneyi*		3		
*P. rufus*	3	2		VU	*Myotis bocagii*	1	3		
***P. seychellensis***		1			*M. goudoti*		2	(+)	
*Rousettus aegyptiacus*	5	41	+		***M. punicus***	1		(+)	NT
*R. madagascariensis*	3	2	+	NT	*M. tricolor*	1	3	+	
***R. obliviosus***		1	+	VU	***M. welwitschii***		1	(+)	
*Scotonycteris zenkeri*^e^	1	1			***Neoromicia bemainty***		1		
*Stenonycteris lanosus*	2	1	+		***N. somalica***		1		
*Epomops* sp.	2				*Nycticeinops crassulus*	1	1		
*Epomophorus* sp.	2	1			*N. schlieffeni*	1	3		
*Megaloglossus* sp.	1				*Pipistrellus* cf*. hesperidus*	1	3		
*Myonycteris* sp.	1				***P. inexspectatus***	1			DD
Pteropodidae sp.	1				*P. kuhlii*^k^	3	2		
**Rhinopomatidae (1/3 species sampled)**					*P. nanulus*	1	3		
***Rhinopoma microphyllum***		1	+		***P. raceyi***		1		DD
**Hipposideridae (11/21 species sampled, plus: 1 genus only)**					*Pipistrellus rusticus*	1	2		
*Doryrhina cyclops*	2	6			*Pseudoromicia brunnea*	1	1		NT
*Hipposideros abae*	2		+		*P. tenuipinnis*	1	6		
*H. beatus*	1	2			*P. tenuipinnis/rendalli*		2		
*H. caffer^g^*	3	7	(+)		*Scotoecus hirundo*	1	1		
*H. fuliginosus^g^*	1	5	(+)		*Scotophilus dinganii*^l^	1	7		
*H. jonesi*	1	1	+	NT	*S. leucogaster*^l^	3	4		
*H. ruber^g^*	3	11	(+)	na	***S. marovaza***		1		
*Macronycteris commersonii*^i^	1	11	(+)	NT	*S. nigrita*^l^	2	3		
*M. gigas*	2	15	(+)		*S. nux*	2			
*M. vittatus*	1	3	(+)	NT	*S. viridis*^l^	2	2		
*Hipposideros caffer/ruber*	1	1			***Vansonia rueppelli***		1	(+)	
*Hipposideros* sp.	3	10			*Hypsugo* sp.		1		
**Nycteridae (7/13 species sampled, plus: 1 genus only)**					*Kerivoula* sp.	1	2		
***Nycteris arge***		1			*Myotis* sp.	1	4		
***N. gambiensis***		1	(+)		*Neoromicia* sp.	2	5		
***N. grandis***		1			*Nycticeinops* sp.		1		
*N. hispida*	2	5			*Pipistrellus* sp.	1	9		
***N. macrotis***		1	(+)		*Scotoecus* sp.	1	4		
*N. major*	1	1		DD	*Scotophilus* sp.	1	4		
*N. thebaica*	4	5	+		Vespertilionidae sp.	1			
*Nycteris* sp.	3	9							
**Megadermatidae (2/2 species sampled)**					**Emballonuridae (7/12 species sampled, plus: 1 genus only)**				
*Cardioderma cor*	1	6	(+)		*Coleura afra*	3	14	+	
*Lavia frons*	2	1			***C. kibomalandy***		1	+	DD
**Rhinonycteridae (5/7 species sampled)**					***Paremballonura tiavato***		1	(+)	
***Cloeotis percivali***		1	+		***Saccolaimus peli***		1		
***Paratriaenops furculus***		1	+		*Taphozous hildegardeae*		2	+	EN
*Triaenops afer*	1	5	(+)		*T. mauritianus*	2	4	(+)	
*T. menamena*		2	+		*T. perforatus*	2	1	(+)	
*T. persicus*		5	(+)		*Taphozous* sp.	1	5		
**Molossidae (26/44 species sampled, plus: 2 genus only, 1 family only)^m^**					**Rhinolophidae (16/36 species sampled, plus: 1 genus only)**				
***Mops aloysiisabaudiae***	1				*Rhinolophus alcyone*	1	3		
*M. ansorgei*		3	(+)		*R. blasii*		2	+	
*M. atsinanana*		2	(+)		*R. clivosus^f^*	2	4	(+)	
***M. brachypterus***	1				***R. damarensis***		1	+	
*M. chapini*		2			*R. darlingi^f^*	1	3	+	
*M. condylurus*	4	14			*R. denti^f^*		3	+	
***M. congicus***		1			*R. eloquens^f^*	1	2	+	
***M. demonstrator***		1			***R. euryale***	1		+	NT
*M. leucogaster*^n^		2			*R. ferrumequinum*	1	1	+	
*M. leucostigma*		2	(+)		*R. fumigatus^f^*	3	1	+	
*M. major*	1	2			*R. hildebrandtii^f^*		6	(+)	
*M. midas*		3			***R. hipposideros***		1	+	
*M. nanulus*		2			*R. landeri^f^*	2	7	+	
***M. niveiventer***		1			*R. simulator^f^*		4	+	
*M. pumilus*	2	15			***R. smithersi***		1	+	NT
*M. pusillus***^o^**		2		VU	***R. swinnyi****^f^*		1	+	
***M. russatus***	1			DD	*Rhinolophus* sp.	3	14		
*M. thersites*		2			**Miniopteridae (15/24 species sampled, plus: 1 genus only)***^h^*				
*Mormopterus acetabulosus*		2	+	EN	***Miniopterus aelleni***		1	+	
*M. francoismoutoui*	1	1	(+)		*M. africanus*		4	+	
*M. jugularis*		2	(+)		*M.* cf. *ambohitrensis*		2	?+	
***Myopterus whitleyi***		1			***M. fraterculus***		1	(+)	
*Otomops madagascariensis*		2	+		*M. gleni*		2	+	
*O. martiensseni***^p^**	3	11	(+)	NT	***M. griffithsi***		1	+	DD
*Sauromys petrophilus*		3	+		*M. griveaudi*		3	+	DD
*Tadarida aegyptiaca*	1	3	+		*M. inflatus*		8	+	
*Mops* sp.	7	6			***M. maghrebensis***	1		+	NT
*Tadarida* sp.	1	1			***M. majori***		1	+	
Molossidae sp.	1				*M. minor*	1	5	(+)	DD
					***M. mossambicus***	1		+	na
**Cistugidae (0/2 species sampled)**					*M. natalensis*		3	+	
**Myzopodidae (0/2 species sampled)**					*M. schreibersii*^j^	2	4	+	(NT/na)
					*M. sororculus*		2	+	
**unidentified `bat’**		10			*Miniopterus* sp.	1	8		

^a^Probably *Epomophorus minor.*

^b^Includes *Megaloglossus azagnyi*.

^c^Often as *Lissonycteris angolensis*.

^d^Includes *Myonycteris leptodon*.

^e^Includes *Scotonycteris occidentalis* and *Scotonycteris bergmansi*.

^f^Might include other *Rhinolophus* spp.

^g^Species group with high cryptic diversity.

^h^High cryptic diversity across genus.

^i^Includes *Macronycteris gigas* and *Macronycteris vittatus*.

^j^Probably including *Miniopterus villiersi*.

^k^As *Pipistrellus deserti*, and as *Pipistrellus aegyptius* (in [29]).

^l^Might include other *Scotophilus* spp.

^m^Taxonomically unresolved.

^n^Probably *Chaerephon pumilus*.

^o^Probably including *Chaerephon pumilus*.

^p^Might include *Otomops harrisoni*

Our analysis revealed biases in sampled species, study sites and data collection. Although some studies aimed to assess viral diversity across multiple taxa, many focused on a subset, including synanthropic species, which may pose a greater risk for viral spillover. Thus, it is not surprising that the gregarious and conspicuous fruit bats *R*. *aegyptiacus* and *Eidolon helvum* were heavily studied (46 and 52 studies). The largest African fruit bat *Hypsignathus monstrosus,* one of three species from which *Orthoebolavirus zairense* (EBOV) RNA has been detected [31] was sampled in 21 studies. The insectivorous free-tailed bat *Mops condylurus* (18 studies), the cave-roosting sheath-tailed bat *Coleura afra*, a large leaf-nosed bat, *Macronycteris gigas* and the small molossid *Mops pumilus* (17 studies each), were also more often sampled than others. Fifty-three species were each sampled only once. When comparing the observed number of studies in which each species (742 species–study combinations) has been reported to the number expected if species (standing richness or sampled richness) were randomly sampled, significant biases were detected (Kolmogorov–Smirnov statistic between observed and expected null distributions, *p* < 0.0001). Fruit bats were overrepresented in the studies we reviewed (35.6%), given that they only represent 13.3% of the 334 recognized African bat species (table 1). Sampling of the cave-dwelling *R*. *aegyptiacus* is explained by its role as a Marburg virus reservoir; this species was lethally sampled in 41 of 46 studies (greater than 8334 individuals). *Eidolon helvum* is tree-roosting and found in large and conspicuous colonies, which may partly explain its disproportionate sampling; 34 of the 52 studies performed lethal sampling (greater than 3992 individuals). High viral diversity has been documented in *E*. *helvum*, including novel viruses with evolutionary relationships to human pathogens (electronic supplementary material, tables S3, S4, S5*b*, S6). Yet, we found no documentation of spillover from this species, and whether comparable sampling effort would detect similar numbers of viruses in other species remains unknown.

Gregarious bat species, including cave-dwelling bats, do not appear to harbour more viruses than other bats [32,33], but might be more likely to share viruses with co-roosting species [33]. Of the 114 studies that provided information on study sites (70.4%), cave and cave-like habitats were preferred and sampled in 66 studies (57.9%). Accordingly, 46.1% of sampled species are cave-roosting. Indeed, caves may be opportunistically targeted for the ease of access to large numbers of bats, which has significant conservation implications given the sensitive nature of cave ecosystems [34]. Disturbance, including bat removal, may have unforeseeable consequences for local cave-dwelling bat colonies and ecosystem functioning, particularly when it leads to abandonment. Cave disturbance can also affect viral transmission dynamics: Marburg virus prevalence in *R. aegyptiacus* increased after a mass extermination triggered by the mineworker fears [35]. In the remaining 48 of the 114 studies that provided site information, bats were captured in forest and savanna habitats, agricultural lands, human settlements and non-natural situations (e.g. animal markets).

**Figure 2.**
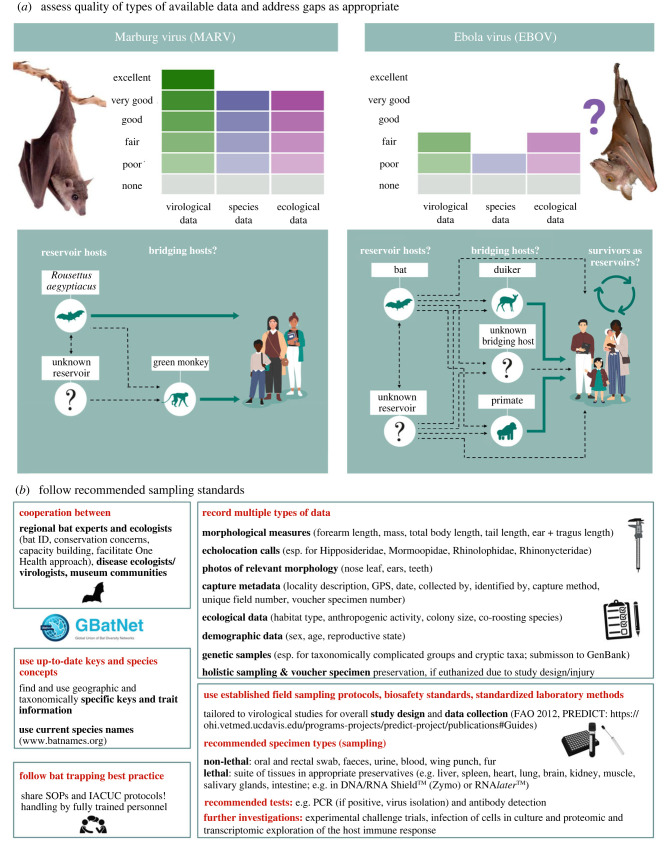
(*a*) Schematic approach to assess available knowledge on specific virus–host relationships (adapted from [47,48]). How well a system has been described is a function of: green: virological data (longitudinal/repeated versus one-off studies; virus isolated/full genomes obtained versus short sequences/inconclusive serology; choice of methods; experimental studies), blue: bat species data (accuracy of species identification; quantity and quality of collected data; sampling strategy), and purple: ecological/environmental metadata (description of habitat, roost site, colony size, life history data); examples shown are for filoviruses, indicating inadequate data on the putative host status of a given bat for EBOV (e.g. for virological data, no EBOV has yet been isolated from any bat, bat species data—confirmation of any bat species as a host is lacking) and high confidence for MARV. See electronic supplementary material, figure S9 for a more schematic illustration allowing qualitative scoring. (*b*) Recommended best practices for bat sampling and identification. Figure created using BioRender.com; *Epomops franqueti* image modified from photo 303584, (*c*) Jakob Fahr, some rights reserved (CC BY-NC), iNaturalist. *Rousettus aegyptiacus* modified from iStock photo.

Data about life history (population estimates, reproductive patterns, age), movement ecology, and habitat use (including co-roosting) are crucial for understanding viral dynamics and the role of bats as reservoir hosts, and for the assessment of spillover risk [36–44]. This information was largely absent in the reviewed studies. Transmission risk may be predicted by ecological data and exacerbated in disturbed habitats [12,45], yet many studies did not report ecological data about captured species (77.8%), or the study site (29.6%). Life-history parameters such as reproductive cycles influence virus infection dynamics, and gradual loss of maternal immunity among young bats increases the number of susceptible individuals [14,15]. Population estimates and identifying the proportion of naive individuals with longitudinal data are thus important to understand viral maintenance and infection dynamics; a recent paper published after our meta-analysis cut-off date that details longitudinal sampling for coronaviruses in *Eidolon helvum* illustrates the strength of this approach [46]. We encourage gathering more comprehensive data on target species and provide a data collection framework (figure 2; electronic supplementary material, figure S9) that will facilitate context-specific goals and priority establishment for bat–virus studies.

### (c) Importance of correct bat species identification

Many authors justify their taxonomic focus by naming ‘bats’ as important sources of emerging diseases. Correct bat species identification is key to reliable and reproducible studies on bat viruses. Assignment to the correct taxonomic suborders (Yinptero- and Yangochiroptera) instead of the still widely used but long-recognized incorrect ‘Mega’- and ‘Microchiroptera’ [49–52]; understanding of the basic phylogenetic relationships of bats, and identification at the species level, are important for any co-evolutionary conclusions about virus transmission between different bat families/species [53,54]. Likewise, correct usage of viral names is important [55], noting that significant viral taxonomic standardization has just occurred, resulting in the adoption of binomial nomenclature and many name changes.

Identifying (African) bats is not trivial due to cryptic diversity and the frequent lack of comprehensive and up-to-date keys. As many as 324 named bat species (and counting) are recognized in Africa [56,57]. In the 15 years since the last benchmark compilation of bat diversity [58], at least 47 species have been described for Africa based on new species discoveries and resolution of high cryptic diversity (e.g. [51,59–64]). Therefore, including descriptions of morphological, ecological, acoustic and genetic traits as necessary to describe a species, should be mandatory in future studies (figure 2). As was demonstrated in the multimammate mouse–Lassa Fever virus system and in the bat clade that includes Rhinolophidae and Hipposideridae, with ties to SARS-related CoVs, taxonomically knowing your host is critically important [54,65].

Forty-six studies in our database (28.4%) did not describe how bats were identified (figure 1*b*). Of the remaining studies, 72 used only morphology (44.4%), 10 only molecular methods (6.2%) and 34 both morphology and genetics (21.0%). Only two of all studies using morphology (1.9%) provided relevant measurements (e.g. forearm length), and 22 studies compared vouchers with museum specimens or cited identification keys (20.8%). The used identification keys, Rosevear [66], Bergmans [67–69] and Patterson & Webala [70], provide a solid basis for morphological identification by experienced scientists, but these keys are partly outdated due to the numerous taxonomic updates since their publication. Because reference sequences are not available for all bat species, and many species-level errors exist in databases such as GenBank [71,72], DNA barcoding alone often does not allow identification to species level [61,63]. Eight of the 29 studies using DNA barcoding only identified specimens that tested positive for viruses (27.6%). Finally, in 128 bat–study combinations (14.7%), bats were identified only to genus (figure 1*b*), which, in fact, is preferable when species identification is uncertain.

Misidentifications and outdated species assignments are likely common in the reviewed papers, but often only evident to bat experts. Yet, species identification is of great importance for follow-up investigations, especially when a particular bat is determined to host a virus of interest. We argue for cross-disciplinary teams between virologists and bat taxonomists, ecologists, regional experts and in-country scientists [73] (often affiliated with natural history museums [74]), to ensure proper species assignment and provide metadata needed for assigning bat–virus relationships (figure 2). If the goals of a particular study require lethal sampling, investigators should adhere to ‘extended specimen’ holistic practices in which multiple types of samples and data are collected for each animal, should deposit their sampled bats in museums where they can be archived in perpetuity [74–77], and should link respective voucher specimens to pathogen studies in museum databases and in publications. In the rare instance that a bat inadvertently dies or is severely injured during sampling intended to be non-lethal, they should be vouchered, increasing the value of the data collected.

### (d) Virus detection methods and associated challenges

Studies in our database focused on detecting RNA/DNA (*n* = 91), antibodies (*n* = 29), or both (*n* = 40; figure 1*d*), providing evidence of exposure to (but not necessarily replication of) a pathogen. Eleven studies detected RNA/DNA with next generation sequencing and 13 attempted virus isolation (figure 1*d*), 10 successfully. Most (96.75%) viral RNA/DNA detections in African bats are based on PCR amplification of specific partial sequences of conserved gene regions [78], which is efficient yet intrinsically biased, and provides little information on formal viral taxonomic placement or on infectivity, virulence or spillover potential. Serology yields higher prevalence as it indicates both current and past infection [5,79], but methods vary widely and cross-reactivity is problematic [16,80–82]. Virus isolation indicates replication and, depending on the sample type, strongly suggests shedding and intra-specific transmission, indicating host competence [83]. Indeed, isolation from non-terminal samples (urine, faeces, saliva, blood), themselves likely spillover routes, provides strong support for reservoir status. However, as illustrated in the filovirus discussion below, viral detection by PCR, serology and viral isolation are but pieces of evidence of variable quality through which host status can be suggested. As recently reviewed by others (e.g. [84–95]), identifying and studying reservoir hosts is not straightforward and ‘target’, ‘source’ and ‘maintenance’ populations may vary. Layered on top of other types of evidence, experimental challenge trials provide the strongest indication that a particular species is a confirmed reservoir host. Additional studies, including infection of cells in culture and proteomic and transcriptomic exploration of the host immune response (e.g. the display of immune tolerance) provide further support for host status and understanding the dynamics of infection [23,95,96].

### (e) Geospatial sampling biases

Geospatial analysis of our dataset demonstrates significant scientific efforts in South Africa, Kenya, Ghana and Gabon (figure 1*e*), with many countries lacking published data. Sheer numbers of bats recorded from each country also varied widely in ways not reflective of relative country size, with the largest number of bats sampled in Gabon (figure 1*f*). Not surprisingly, bat viral survey locations appear to be at least practically driven by geopolitical and capacity considerations. At the family, genus, and especially species level, bat biodiversity in Africa is highly concentrated in equatorial, tropical sub-Saharan Africa [56]. Our geographical analysis (figure 1*g*) demonstrates that greater effort is required in many countries, especially those with zero sampling effort to date.

## 3. African bats and virus families important to human health

Viruses with clear importance to human health are generally clustered within four viral families: *Corona-, Paramyxo-, Rhabdo-* and *Filoviridae*, although evidence for viruses in many other lineages exists (table 2; electronic supplementary material, tables S3 and S8). Definitive evidence for direct spillover from bat to human or spillover via a bridging host [152] between African bats and humans only exists for Sosuga, Marburg and Duvenhage virus. For the majority of viruses detected in African bats (22% of bat viral sequences worldwide through 2020), there is no documented human infection. Recent modelling studies have shown sampling effort to be the most important predictor of bat infection [91,153] (electronic supplementary material, text S6), reminding us that the absence of evidence may be heavily biased by which species are sampled.

**Table 2.** Zoonotic viruses with known or potential relation to human disease and evidence for a bat origin or potential relation to African bats.

virus family	virus genus/ subfamily	virus	number of human infections/fatalities	zoonotic source of human infection	virus evidence from bats	bat–human transmission shown	references
*Coronaviridae*	*Alphacoronavirus*	HCoV-229E	unknown, mostly mild respiratory disease	possibly camelids (endemic in humans)	possible evolutionary origin in hipposiderid bats (PCR evidence)	no	[97–103]
	*Alphacoronavirus*	HCoV-NL63	unknown, mostly mild respiratory disease	unknown (endemic in humans)	possible evolutionary origin in rhinonycterid or hipposiderid bats (PCR evidence)	no	[98,100,104]
*Filoviridae*	*Orthomarburgvirus*	Marburg virus	498/397 (1967 −2023)	non-human primates, *R. aegyptiacus*	*R. aegyptiacus,* considered as reservoir host based on PCR and virus isolation	yes	[15,17,18,21,22,26,105–107]
	*Orthoebolavirus*	Ebola, Bundibugyo, Sudan, Tai Forest virus	34 849/15 343 (1976–2023)	non-human primates, duiker, possibly bats	natural reservoir unknown; PCR positives (*E. franqueti*, *H. monstrosus, M. torquata*), serological evidence from several African bats	no	[21,31,80,82,105,108–122]
*Flaviviridae*	*Orthoflavivirus*	Dengue-2 virus	estimated 400 million per year/40 000 per year	mosquitoes	serological evidence from *M. pumilus*, *M. condylurus, E. labiatus*	no	[123,124]
	*Orthoflavivirus*	West Nile virus	56 569/2773 (1999–2022)	mosquitoes	serological evidence from *E. helvum* and *E. labiatus*	no	[124,125]
	*Orthoflavivirus*	Yellow fever virus	estimated 200 000 per year/30 000 per year	mosquitoes	serological evidence from *R. aegyptiacus*	no	[124,126]
*Nairoviridae*	*Orthonairovirus*	Crimean Congo haemorrhagic fever virus or CCHF-like viruses	estimated 10 000–15 000 per year/500 per year	ticks, livestock	serological evidence in 10 African bat species for CCHFV or a closely related virus belonging to the CCHFV serotype	no	[127–131]
	*Orthonairovirus*	Dugbe virus	unknown, moderate clinical manifestation	ticks, livestock	serological evidence from *C. afra*	no	[131,132]
*Orthomyxoviridae*	*Alphainfluenzavirus*	Avian influenza A (H9)	H9N2 caused > 100 infections and small number of deaths	poultry	serological evidence for avian influenza H9 in *E. helvum*	no	[133–135]
*Paramyxoviridae*	*Orthorubulavirus*	Sosuga virus	1/0	*R. aegyptiacus*	*R. aegyptiacus* (PCR evidence)	yes	[136]
	*Pararubulavirus*	Achimota virus 1 & 2	3 of 443 seropositive for AchPV2	unknown	serological evidence for both viruses in *E. helvum*	no	[137]
*Phenuiviridae*	*Phlebovirus*	Rift Valley fever virus	4641/957 (2000–2016); no systematic surveillance	mosquitoes, livestock	serological evidence from *R. aegyptiacus*, *E. labiatus*, virus isolation from *L. frons*, *H. caffer*, *Myotis* sp.	no	[124,131,138,139]
*Rhabdoviridae*	*Ledantevirus*	Kumasi rhabdovirus	6 of 163 seropositive	possibly *E. helvum*	KRV isolated from *E. helvum*	possibly	[140]
	*Lyssavirus*	Mokola virus	2/2	unknown	never isolated from bats; serological evidence uncertain (cross reaction with Lagos bat virus); other potential reservoirs are African shrews and insectivorous rodents	no	[141–146]
	*Lyssavirus*	Duvenhage virus	3/3	African bats	African bats (e.g. virus isolation from *N. thebaica)*	yes	[141,147–150]
*Spenareoviridae*	*Orthoreovirus*	Pteropine orthoreovirus	infections may be common in Southeast Asia, no known deaths	Asian *Pteropus* sp.	Asian *Pteropus* sp., African *Myonycteris angolensis ruwenzorii* (PCR evidence)	yes	[151]
*Togaviridae*	*Alphavirus*	Babanki virus	unknown	mosquitoes	serological evidence from *E. labiatus, R. aegyptiacus*	no	[124]

The 24 African coronavirus studies we analysed reported RNA sequences related to human-relevant viruses, i.e. SARSr-CoV, MERSr-CoV, HCoV-NL63 and HCoV-229E (table 2; electronic supplementary material, text S3, table S3). Paramyxovirus RNA was detected in 45 African bat species (electronic supplementary material, text S4, table S4*a,b*). Nine studies reported potential zoonotic *Orthoparamyxovirinae* members, mainly in fruit bat species and based on serology or RNA sequences, with no documented spillover. Four studies detected sequences related to human mumps and to parainfluenza virus 2 and 4. Within this same viral subfamily (*Rubulavirinae*), Sosuga virus was initially isolated from a wildlife biologist and subsequently identified by PCR and full genome sequencing from *R. aegyptiacus* [136,154], whose host status is strongly supported by experimental infection studies [155]. *Rabies virus* (RABV) and the 16 rabies-related *Lyssavirus* species in the family *Rhabdoviridae* cause the fatal disease rabies. Due to very low RNA detectability outside of brain tissue, most surveillance studies prefer serology (electronic supplementary material, text S5, table S5*a,b*). In Africa, rabies-related lyssaviruses have each generally been associated with a different bat species, yet knowledge on lyssavirus epidemiology and ecology in African bats is highly limited [156].

The greatest attention to bat viruses in Africa has focused on filoviruses. Of the four genera encountered in bats, only two occur in Africa (*Orthoebolavirus* and *Orthomarburgvirus*). *Orthomarburgvirus* is restricted to Africa and contains Marburg (MARV) and Ravn virus. *Orthoebolaviruses* occur in Africa and Southeast Asia (electronic supplementary material, text S6, table S6*a–d*). They include six species: EBOV, Bundibugyo, Sudan, Taï Forest, Reston and Bombali [157]. Both the orthomarburg- and several orthoebolaviruses cause high mortality rates in humans (table 2). In our dataset, sampling for the *Filoviridae* was done in 23.5% (*n* = 38) of the 162 studies, and accounted for nearly one-third (29.0%) of all confirmed captures (*n* = 24 875) and almost half of lethally sampled bats (*n* = 19 089). Sampling efforts for orthomarburg- (*n* = 17 750) and orthoebolaviruses combined (*n* = 18 574) are similar, but the results and associated information content could hardly differ more (figure 2*a*; electronic supplementary material, text S6).

### (a) Contrasting examples: information on bats as reservoir hosts of Marburg and Ebola virus

The first detection of MARV RNA and antibodies in bats [158] was followed 2 years later by virus isolation from *R. aegyptiacus* [17]; epidemiological ties to caves directed early efforts towards this populous cave-dwelling bat [28]. Isolation has subsequently been successfully repeated [13,14,18,26,35,159]. That *R*. *aegyptiacus* is a reservoir host of MARV [159] is based on: (1) high genetic similarity between bat and human virus isolates (99.3%;[13]); (2) serological findings of MARV-specific antibodies [17,21,22,159]; (3) seasonal infection peaks that match spillover to humans [14,19], and (4) experimental infection of *R*. *aegyptiacus* without overtly apparent symptoms [15,159], with a transcriptional host response that indicates immunological tolerance [23], and with oral, faecal and urine virus shedding [15,24,159–161], likely the spillover route [20,25]. The relation between MARV and *R. aegyptiacus* as a reservoir host is based on high-quality virological, species and ecological/environmental information content (figure 2*a*), and has the highest scientific support of all bat–virus relationships in the 162 reviewed papers. Nevertheless, several other bat species also tested positive for MARV (electronic supplementary material, table S6*b–d*; [21,22,80,159]), interpreted as incidental spillover between bat species [159]. Given that PCR and serological evidence for MARV exists in two insectivorous bat species (*Miniopterus* *inflatus* and *Rhinolophus eloquens*) (electronic supplementary material, table S6*b*; [22]), further research on the role of these and other (likely cave-roosting) species is needed.

By contrast, *Orthoebolavirus* nucleic acid detections in bats are rare, with no virus isolation (figure 2*b*; electronic supplementary material, text S6, table S6*c*). Efforts have largely focused on EBOV within fruit bats (based upon early detection in *Epomops franqueti*, *Hypsignathus monstrosus*, and *Myonycteris torquata* [31,162]), with additional sampling in a variety of other bats and vertebrates [91,116,157,163]. A partial EBOV genome was reported in 2019 in the popular press from *Miniopterus inflatus* in Liberia [164] (which is likely the West African *M*. *nimbae*, newly described several months later [165]). Remarkably, RNA detection of the recently discovered Bombali virus [157], which is probably not human-relevant [166,167], has been PCR-confirmed five times [168–171] in two species of free-tailed bats (erroneously ‘fruit bats’ in [167]) across four countries. Other links between bats and orthoebolaviruses are largely based on serology (electronic supplementary material, table S6*d*; [163,172,173]).

Early experimental infection studies with EBOV demonstrated replication with seroconversion in all three bat species tested (*Mops condylurus*, *M*. *pumilus* and *Epomophorus wahlbergi*), with viral shedding in the faeces in *E. wahlbergi* [174]. Infection of *R. aegyptiacus* with five viruses in the genus *Orthoebolavirus* (except Bombali) resulted in injection-site replication only, except for Sudan *virus*, which replicated without shedding [160]; *R. aegyptiacu*s is a dead-end host for these orthoebolaviruses. Additional evidence for bats as reservoir hosts for orthoebolaviruses comes from cell line studies, which suggest differential susceptibility of bat species to infection [175,176], including *E. helvum*, whose cells are refractory to viral entry [175,177]. In particular, immune tolerance of EBOV and Bombali virus has been documented in *Mops condylurus* [176,178,179], which displays little histopathology when infected [171]. This body of evidence, along with modelling studies (electronic supplementary material, text S6), point to forest-dwelling bats as likely *Orthoebolavirus* reservoir hosts. However, more recent studies indicate a more complex scenario with potential bridging hosts and environmental influences, highlighting the need for multidisciplinary approaches [153,180–189]. Direct transmission of EBOV from bats to humans has been posited in two theoretical scenarios [112,162], without evidence. By contrast, there is convincing evidence for EBOV spillover from symptomatic great apes and potentially duikers to humans [190–194] (figure 2*a*; electronic supplementary material, text S6). Importantly, spillovers have exclusively been documented within the distribution ranges of chimpanzees and bonobos (electronic supplementary material, text S6, figure S6), which may have had contact with the natural reservoir(s) of EBOV [195,196] when feeding at the same fruit trees, through consuming infected animals [197] or even through contact with aquatic or semi-aquatic reservoir hosts [198]. Molecular evidence from recent EBOV outbreaks suggests a human survivor origin rather than zoonotic transmission [199,200], raising the possibility that some previous outbreaks (including the 2013 West African outbreak) are not from spillover [201]. Recent findings continue to support bats as key players in the EBOV story, but the epidemiology is complex and many gaps remain in our understanding (figure 2*a*; electronic supplementary material, text S6).

## 4. Communication of virological findings in bats and conservation implications

‘When a man is hated in the village, he will be accused of raising dust even when he jumps into a pool of water’ - Ugandan proverb

Bats are important ecosystem service providers and many, including African species, are under significant threat (e.g. habitat encroachment and loss, degradation, hunting, etc.) [9,10,202]. Bats are already widely perceived to be dangerous [203] and the perception that spillover from bats poses a significant risk to humans increases the threat of culling or roost site destruction. For example, portrayal of ‘bats’ as a definitive spillover source of orthoeboloaviruses is common across the surveyed literature, with consequences for public perception, conservation, as well as other research sectors [204] and during outbreaks, epidemiological messaging runs the risk of characterizing bats as ‘epidemic villains' [204–206].

Precision and tone of language and data interpretation are critical, especially expressions such as ‘public health concern’, ‘threat to humans’, ‘spillovers with fatal consequences', or ‘reservoirs of many recently emerged zoonotic viruses’, all of them present in the reviewed literature. At times, indirect evidence was used to infer scenarios and in the most extreme cases, authors linked viruses to bats simply based on their presence in an area [162,207]. In our reviewed papers, 53.1% of the abstracts explicitly framed bats as a threat to human wellbeing; only 19.1% explicitly stated the contrary and the rest made no statement. This pattern recurred in the discussions (dangerous: 62.3%, non-dangerous: 0.8%). Potential transmission pathways were rarely specified in the abstract (6.1%), and only occasionally addressed in the discussion (32.1%). Numbers of human deaths were only reported in a single abstract and 7.4% of discussions, and ecosystem services barely at all (three abstracts and 4.9% of discussions). Only one study expressed concern about misguided consequences for bats.

The strengths and weaknesses of specific scientific findings are difficult to understand for scientists, and even more so for the public. Disease-related speculation not supported by strong evidence but shaped by bat–virus catchphrases lacking scientific integrity undermines decades of conservation efforts [208]. Careful, scientifically correct wording is crucial for how results are disseminated to and by the press [209,210], informing the press and the public about the relation of bats and viruses based on scientific information content, while considering the challenges of contextualizing scientific findings from a non-expert audience perspective. Fortunately, efforts aimed at mitigating bat–human interactions can successfully balance bat conservation and human health [159]. Indeed, conservation efforts that target habitat preservation are linked to spillover prevention [12,211–213]. Balanced messaging to prevent extirpation and promote conservation needs to be delivered through awareness raising campaigns, targeting and led by local communities and authorities [155,214–220].

## 5. Conclusion and recommendations

Despite the large body of the literature and intensive research efforts, evidence for links between African bats and human-relevant disease is sparse. Few examples of bat surveillance efforts translate into the frequently declared goal, i.e. the prediction or prevention of spillover. Considering the risks emerging zoonotic diseases pose, a taxonomically broad One Health approach at the human–animal–ecosystem interface, with multidisciplinary and local team members, should be deployed (figure 2*b*). Efforts to identify potential bridging hosts of bat-borne viruses and to identify human behaviour that fosters spillover are needed. Additionally, it is crucial to explicitly distinguish between the evolutionary origin of a virus (e.g. *Betacoronaviruses* in bats), and the actual reservoir and/or spillover source. Current global initiatives (e.g. the Global Union of Bat Diversity Networks [221]) are actively working to strengthen, standardize and share research protocols and can connect non-bat experts with potential collaborators around the world. For host–virus systems identified for further study based upon human health risk or other research priorities, assessing the quality and types of available virological, species, and ecological data will facilitate the identification of knowledge gaps and direct subsequent efforts to fill those gaps. Attending to and prioritizing the bat conservation implications of bat–virus studies and the sociological elements at the bat–human interface will be crucial for continued studies of potential zoonoses within the One Health context.

## Data Availability

Data used in this study are available from the Dryad Digital Repository: https://doi.org/10.5061/dryad.c866t1gcx [222]. Supplementary material is available online [223].
